# Supplementary material for: End Sequence Analysis Toolkit (ESAT) expands the extractable information from single-cell RNA-seq data
Source: Genome Res. 2016 Oct;26(10):1397–410. doi: 10.1101/gr.207902.116 (PMC5052061; doi:10.1101/gr.207902.116)
Supplement: Supplemental Material [file supp_gr.207902.116_Supp_Materials.tar.gz › ESAT-esat_v0.1/doc/html/classjaligner_1_1formats_1_1_format.html]

ESAT: jaligner.formats.Format Class Reference


ESAT


- Main Page
- Related Pages
- Packages
- Classes
- Files

- Class List
- Class Index
- Class Hierarchy
- Class Members

All Classes Namespaces Files Functions Variables Enumerator Pages

- jaligner
- formats
- Format

Public Member Functions |
List of all members

jaligner.formats.Format Class Referenceabstract

Inheritance diagram for jaligner.formats.Format:

[legend]

|  |  |
| --- | --- |
| Public Member Functions | |
| abstract String | format (Alignment alignment) |
|  | |
| void | setId (String id) |
|  | |
| String | getId () |
|  | |

## Detailed Description

Abstract format

Author
:   Ahmed Moustafa (ahmed.nosp@m.@use.nosp@m.rs.sf.nosp@m..net)

## Member Function Documentation

|  |  |  |  |  |  |
| --- | --- | --- | --- | --- | --- |
| abstract String jaligner.formats.Format.format | ( | Alignment | *alignment* | ) |  |

abstract

Formats alignment

Parameters
:   |  |  |
    | --- | --- |
    | alignment |  |

Returns
:   formatted alignment

See also
:   Alignment

|  |  |  |  |  |
| --- | --- | --- | --- | --- |
| String jaligner.formats.Format.getId | ( |  | ) |  |

Returns format id

Returns
:   id

|  |  |  |  |  |  |
| --- | --- | --- | --- | --- | --- |
| void jaligner.formats.Format.setId | ( | String | *id* | ) |  |

Sets format id

Parameters
:   |  |  |
    | --- | --- |
    | id | to set |

Here is the caller graph for this function:

---

The documentation for this class was generated from the following file:

- java/jaligner/formats/Format.java


---

Generated on Fri Aug 15 2014 15:11:06 for ESAT by  

 1.8.7
